# Modeling the Agility MLC in the Monaco treatment planning system

**DOI:** 10.1120/jacmp.v17i3.6044

**Published:** 2016-05-08

**Authors:** Michael Snyder, Robert Halford, Cory Knill, Jeffrey N. Adams, Todd Bossenberger, Adrian Nalichowski, Ahmad Hammoud, Jay Burmeister

**Affiliations:** ^1^ Department of Oncology Wayne State University School of Medicine Detroit MI USA; ^2^ Department of Radiation Oncology Karmanos Cancer Institute Detroit MI USA

**Keywords:** MLC modeling, IMRT commissioning, Monaco

## Abstract

We investigate the relationship between the various parameters in the Monaco MLC model and dose calculation accuracy for an Elekta Agility MLC. The vendor‐provided MLC modeling procedure — completed first with external vendor participation and then exclusively in‐house — was used in combination with our own procedures to investigate several sets of MLC modeling parameters to determine their effect on dose distributions and point‐dose measurements. Simple plans provided in the vendor procedure were used to elucidate specific mechanical characteristics of the MLC, while ten complex treatment plans — five IMRT and five VMAT — created using TG‐119‐based structure sets were used to test clinical dosimetric effects of particular parameter choices. EDR2 film was used for the vendor fields to give high spatial resolution, while a combination of MapCHECK and ion chambers were used for the in‐house TG‐119‐based procedures. The vendor‐determined parameter set provided a reasonable starting point for the MLC model and largely delivered acceptable gamma pass rates for clinical plans — including a passing external evaluation using the IROC H&N phantom. However, the vendor model did not provide point‐dose accuracy consistent with that seen in other treatment systems at our center. Through further internal testing it was found that there existed many sets of MLC parameters, often at opposite ends of their allowable ranges, that provided similar dosimetric characteristics and good agreement with planar and point‐dose measurements. In particular, the leaf offset and tip leakage parameters compensated for one another if adjusted in opposite directions, which provided a level curve of acceptable parameter sets across all plans. Interestingly, gamma pass rates of the plans were less dependent upon parameter choices than point‐dose measurements, suggesting that MLC modeling using only gamma evaluation may be generally an insufficient approach. It was also found that exploring all parameters of the very robust MLC model to find the best match to the vendor‐provided QA fields can reduce the pass rates of the TG‐119‐based clinical distributions as compared to simpler models. A wide variety of parameter sets produced MLC models capable of meeting RPC passing criteria for their H&N IMRT phantom. The most accurate models were achievable using a combination of vendor‐provided and in‐house procedures. The potential existed for an over‐modeling of the Agility MLC in an effort to obtain the fine structure of certain quality assurance fields, which led to a reduction in agreement between calculation and measurement of more typical clinical dose distributions.

PACS number(s): 87.56.nk, 87.53.Kn, 87.55.km, 87.55.Qr

## I. INTRODUCTION

The Agility multileaf collimator (MLC) (Elekta, Stockholm, Sweden) contains 160 leaves that can be interdigitated to deliver complex IMRT and VMAT plans.^(1)^ To achieve accuracy in dose calculation for these IMRT and VMAT plans, the Monaco 5.0 treatment planning system (Elekta Oncology Systems, Crawley, UK) uses a tunable MLC model to best fit the true exit fluence from the linac head. Due to very small differences in the installation and setting of the MLC these parameters are necessarily linac‐specific. To determine the parameters that best fit an individual installation, the vendor provides a set of predesigned fields referred to as the ExpressQA package.^(2)^ These fields are offered to the physics staff in an effort to provide a means to quickly determine the most appropriate set of MLC parameters by comparing Monaco‐calculated dose distributions with those measured at the machine. This process can be completed in tandem with the help of the vendor, or, alternatively, the local physics staff can follow the procedures for adjusting the MLC parameters in‐house.

The procedures associated with the vendor‐supplied quality assurance (QA) package generally suggest abiding the defaults for all but a single parameter labeled “leaf offset.” This parameter is meant to define the physical deviation from the calibrated “zero position” of the MLC leaves that might occur during the head installation process. Setting a negative value for this number effectively tells the planning system that a set gap between two opposing leaves is slightly smaller physically than the stated gap, whereas setting a positive value indicates to the planning system that the same gap is, in reality, slightly larger physically that what is being set in the planning system. As a result, this leaf offset parameter controls the effective output factor for very small apertures associated with dynamic IMRT and VMAT plans, and can considerably affect the absolute calculated dose.

Although the leaf offset is the primary parameter discussed in the vendor‐supplied procedures, the model itself contains many more user‐adjustable parameters.^(3)^ The values of these other parameters should be discernible by further use of the vendor‐supplied fields with careful high‐resolution dose distribution measurements. However, it is noteworthy that none of the procedures outlined in the vendor modeling package include point‐dose measurements with calibrated ion chambers. Instead, dose distribution evaluations are relied upon exclusively, with an emphasis placed on the proper calibration of the equipment chosen to measure said distributions.

Our in‐house methodology for tuning the MLC model of other treatment planning systems includes a test of both point‐dose and planar dose accuracy, using plans created for the varied anatomical sites provided by the AAPM TG‐119 report.^4^, ^5^, ^6^, ^7^, ^8^ Due to the lack of point‐dose measurements in the vendor‐supplied package and the relatively small number of fields present, it was concluded that we would first follow the vendor‐supplied modeling procedure, and then supplement this procedure with a variation of our own TG‐119‐based procedures, obtaining various MLC parameter sets at each stage. In this manuscript we submit our experience with the adjustment of the MLC parameters using these procedures individually and collectively, provide in‐house measured distribution and point‐dose data, and finally present the results of external review and credentialing for several potential MLC models.

## II. MATERIALS AND METHODS

The MLC modeling began with the use of a vendor‐assisted modeling procedure. The QA fields provided by the vendor in the ExpressQA package are delivered by the user to a measurement device of the user's choice, and the results are shared with the vendor. The vendor then uses the results to create an MLC model which is sent back to the user for inclusion in the planning system. After this procedure was completed and our model was in hand, in‐house testing commenced through the use of the ExpressQA package and our own methodologies involving TG‐119‐based planning and delivery. A series of MLC modeling parameter sets were generated, broken into complexity classes. A subset of MLC models considered clinically viable was sent — along with the model provided by the vendor procedure — for external evaluation using the Imaging and Radiation Oncology Core (IROC) H&N phantom.^(9)^

### A. Vendor‐evaluated ExpressQA

The ExpressQA package consists of eight QA fields. Of these eight fields, two represent simulated H&N ports to provide some means of testing pseudoclinical performance, and two are simple open fields to check symmetry. Four fields remain for discerning specific dosimetric features of the MLC: 3ABUT — a simple step‐and‐shoot plan where three consecutive segments are matched to create a uniform field; DMLC1 — a dynamic sweep field designed to produce the same distribution as the 3ABUT given perfect MLC calibration; 7SEGA — a matching field similar to the 3ABUT with narrower segments; and finally FOURL — a four‐segment, step‐and‐shoot port that consists of progressively smaller nested L‐shapes designed to test tongue‐and‐groove effects and MLC transmission, among other things.

To initiate the vendor‐evaluated package, all eight fields were delivered to a Sun Nuclear ArcCHECK (Sun Nuclear Corporation, Melbourne FL, USA) — one of the QA devices explicitly called out as appropriate in the vendor instructions. The measurements were subsequently sent to the vendor, whereupon comparisons were made between Monaco calculated distributions and those measured with the ArcCHECK. An MLC model was tuned by the vendor, and the parameters were installed in our own planning system.

### B. In‐house ExpressQA utilization

Upon receipt of the vendor‐tuned MLC model, we proceeded to perform our standard test of the planning system through the planning and delivery of a variety of simulated treatments based on those found in the report of TG‐119. It was noted immediately that the predicted point doses of the model were deviating, on average, more than we would expect given our historical results. We therefore began recalculating the ExpressQA package in‐house in an attempt to fine‐tune the MLC parameterization. Our in‐house experience revealed an inherent difficulty in the interpretation of ArcCHECK measurement and calculated dose comparisons for the ExpressQA fields. This difficulty was initially thought to be due principally to spatial resolution issues (further discussion of this appears below). This led to the use of EDR2 film as the in‐house QA device of choice for these fields. Emphasis was placed on the 3ABUT and FOURL fields to determine MLC parameters, with the DMLC1 field used to a lesser extent to help diagnose appropriate values for interleaf leakage.

Table 1 displays the parameters considered in the modeling presented here. There are more user‐adjustable parameters in the MLC model; however, all other parameters either had a much smaller effect on the calculated distributions or were associated with the diaphragms. No parameters were changed for the diaphragm model, and no parameters were changed to model backscatter into the monitor chamber.

Monaco is a Monte Carlo–based treatment planning system which requires the user to adjust allowable statistical uncertainty and voxel size. For the evaluation of the vendor QA fields, a statistical uncertainty of 1% per plan and a voxel size of 2 mm were chosen. For the most part, tests of smaller voxel sizes and statistical uncertainties show no differences in the discernible structure of the dose distributions. The only exception occurred when modeling what the vendor refers to as corner leakage. For this portion of the modeling, the 3ABUT plan was calculated at a 1 mm grid size with 0.5% statistical uncertainty.

**Table 1:** List of specific parameters for each MLC model.

	*Leaf Tip Leakage*	*Leaf Offset*	*Leaf Transmission*	*Groove Width*	*Interleaf Leakage*	*Corner Leakage*
Vendor	1.16	0.05	^a^	^a^	^a^	^a^
A	1.13	‐0.1	^a^	^a^	^a^	^a^
B	1.25	‐0.32	^a^	^a^	^a^	^a^
C	1.07	0.0	0.0063	^a^	^a^	^a^
D	1.03	0.05	0.0072	^a^	^a^	^a^
E	1.10	‐0.30	0.006	1.0	5.0	1.0
^a^	1.10	0.0	0.005	0.4	3.0	0.0

a Denotes the default value defined as the value present in the MLC Parameter .xml file after vendor beam‐modeling but before vendor MLC‐modeling. These values may be user/energy‐specific if adjusted by the vendor during beam‐modeling.

### C. TG‐119 usage

In addition to the in‐house usage of the ExpressQA package, pseudoclinical plans were also created for a set of structures and dose constraints adapted from the TG‐119 report. With these plans, direct point doses could be measured in phantom to tune not only distribution metrics but also absolute doses both in and out of target.

The downloadable phantom provided with the report from TG‐119 was used as the patient for all plans produced in the study. The structure sets used for planning were combination of those included in TG‐119 and custom structures that are intended to provide more realistic anatomical cases for both IMRT and VMAT commissioning and validation.^(10)^ The structure sets used in this study were: i) prostate* ii) C‐shape hard*, iii) prostate and lymph nodes, iv) head and neck*, v) and head and neck with simultaneous integrated boost (structure sets from the original TG‐119 sets marked with *). Treatment plans were created for each structure set using both IMRT and VMAT techniques optimized to plan objectives included in the TG‐119 report as well as objectives based on clinically realistic goals for the given custom treatment type.^(10)^ A conventional, flattened 6 MV beam was used for all plans. The treatment plans were optimized using the default MLC model parameters (Table 1). These plans were then used in the subsequent model comparison by maintaining the control points and monitor units in the original optimized plans, and recalculating the dose using updated MLC modelling parameters.

For the TG‐119 portion of this study we used a dose grid resolution of 3 mm, with a statistical uncertainty set to 1% per plan. Both of these values were chosen due to our intent to use these same planning parameters in clinical use. It is possible to create plans of finer resolution and lower noise, but in our current experience such plans would be difficult to produce in a clinically feasible time frame (~ 1 hr, including optimization and dose calculation), and it was deemed inappropriate for this section of the modeling to use planning parameters that would not be employed clinically.

In Monaco it is possible to set preoptimization limits on the number and size of segments allowed during plan optimization. For the IMRT plans created in this study the total number of segments allowed for an individual beam was set to 30, with a minimum segment width set to 5 mm. For the VMAT plans the total number of segments allowed in an individual arc was limited to 120, with a minimum segment width of 10 mm.

The dose calculation algorithm in Monaco reports dose‐to‐medium by default, with the option to convert the reported value to dose‐to‐water. It has been noted that this conversion may introduce uncertainty into the reported dose,^(11)^ so in order to remove any such uncertainty the electron density (relative to water) of the phantom in the planning system was forced to a value of 1.0, resulting in the reporting of dose‐to‐medium where that medium was in fact simply water.

Once acceptable plans were created, they were recalculated on a simulated QA water phantom to allow evaluations of planar dose distributions using Sun Nuclear MapCHECK.

All fields — both IMRT and VMAT — were set to gantry 0° and recalculated to a depth of 5 cm in the phantom. A discussion of this choice of distribution evaluation follows below.

The plans were exported to the record‐and‐verify system and delivered using a Versa HD (Elekta Oncology Systems) linear accelerator fitted with an Agility treatment head.^(1)^ The phantom from the TG‐119 structure sets was recreated using a stack of solid water with the inclusion of a 2 cm thick plate drilled to allow the insertion of a 0.3 cc PTW 30010 ion chamber (PTW Freiburg GmbH, Freiburg, Germany). All measurements were calibrated using the methods described in TG‐119 involving the association of a measured charge at the center of the phantom due to the delivery of an AP/PA beam combination to that calculated using the same setup in the planning system.

In addition to point doses measured in the solid water stack, plans were also delivered to the Sun Nuclear MapCHECK QA device. IMRT plans were delivered and measured per‐beam, whereas the VMAT plans were measured over a complete arc. All plans, both IMRT and VMAT, were delivered at gantry 0° to remove mechanical gantry sag effects and specifically highlight MLC model deviations.

### D. MLC model adjustment and external evaluation

The vendor‐provided QA fields were evaluated using EDR2 film scanned using a VIDAR scanner (VIDAR Systems Corporation, Herndon, VA). The images were imported into RIT113 software (OSL Oncology Systems Limited, Shrewsbury, UK) and calibrated using a standard step‐wedge calibration procedure. Using a small MATLAB script (MathWorks, Inc., Natick, MA), the dose planes from Monaco were converted into RIT MATLAB files for import and comparison. The 3ABUT field was used to investigate changes to the leaf offset, leaf tip leakage, leaf transmission, and corner leakage parameters. The FOURL field was used to investigate changes to the leaf transmission and groove width parameters. And finally, the DMLC1 field (and slight modifications thereof) was used to investigate changes to the interleaf leakage parameter.

For the IMRT and VMAT plans delivered to the solid water stack, point‐dose measurements were compared to mean dose values taken averaged over the volume of the ion chamber in the planning system dose distribution. This alleviated some of the statistical noise in the plan and provided a better representation of the predicted measureable dose. Plans were recalculated for a number of parameter combinations in an attempt to match point‐dose measurements on average throughout all plans.

The planar dose comparisons were made by exporting dose plane information from Monaco and using Sun Nuclear's patient software to evaluate any discrepancy seen in the measured distribution. Gamma analysis was performed using 2%/2 mm criteria, where the dose is evaluated with respect to the global maximum, and a minimum threshold of 10% of that global maximum is used for inclusion of a data point within the analysis.

Once a broad investigation of the parameters was concluded, a subset of all potentially clinically viable parameter‐sets — including three in‐house parameter‐sets and the parameterset provided by the vendor, for comparison — was used to recalculate a previously optimized H&N IMRT plan for the IROC H&N phantom. A comparison was then made between the point dose and distribution measured by the external accrediting body and the four planned dose matrices. As with the above TG‐119 comparisons, any differences seen can be directly attributable exclusively to the MLC model parameters, as each planned dose distribution used the same optimized MLC segments.

## III. RESULTS

The vendor procedure for creating an MLC model simply returns an MLC parameter set that is inserted into the planning system via transfer of an .xml file. The relevant parameters from the vendor set are listed in Table 1. Throughout the rest of the results, all calculations done using this parameter set are labeled “Vendor”. Also in Table 1 are five other parameter sets labeled A through E. These parameter sets are of increasing complexity with respect to the number of parameters adjusted from default, and have been chosen from a large pool of tested sets to display specific consequences of parameter set choice. Parameter Sets A and B restrict changes from default to only the leaf offset and leaf tip transmission parameters. Set A was chosen to represent the situation where the parameters were kept as close to the defaults as possible while still producing reasonable point dose and QA plan matching, while Set B is a set chosen to display the ability of parameter values at the extremums to offset one another. Sets C and D allow leaf transmission changes from default, with Set C presenting values closer to default and Set D allowing an “unrealistic” value of leaf tip transmission to be offset by both leaf offset and leaf transmission. Finally, parameter Set E allows all parameters in the model to deviate from default to any degree in order to best match a combination of the point‐dose measurements, planar measurements of the clinical plans, and profile measurements of the vendor‐provided QA plans.

**Figure 1:**
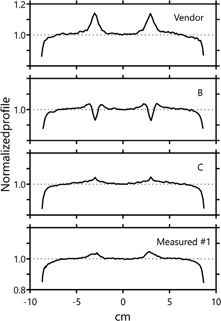
Normalized profiles taken across the dose distribution for the 3ABUT plan. The top three profiles represent plans calculated using different MLC models. The bottom profile was measured using EDR2 film. Profiles were taken through isocenter. Table 1 displays the parameters associated with each model.

To demonstrate the impact of the choice of particular leaf offset and leaf tip transmission combinations, profiles taken across the 3ABUT field are plotted for distributions calculated using three different MLC models in Fig. 1. Also displayed is a profile taken across the 3ABUT measured distribution. The profiles were all taken through isocenter, parallel to the leaf motion. As can be seen in the figure, the measured profile exhibits small maxima at the match‐lines of the 3ABUT segments. As instructed by the ExpressQA documentation, this same topology can be created in the calculated distributions by setting positive leaf‐offset values, which simulates the situation where a small gap exists at the supposed match‐line in the plan. This same effect can also be created in the distribution by leaving the leaf offset parameter at 0 or at a small negative value, and increasing the leaf tip leakage parameter to effectively create a region of lesser beam attenuation, as opposed to a physical gap due to the position of the leaves. The vendor‐provided model creates an exaggerated version of the measured topology, whereas model B creates a direct inverse of the topology, suggesting an inaccurate MLC model.

**Figure 2:**
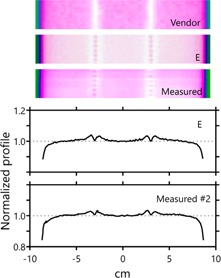
Additional normalized profiles taken across the dose distribution for the 3ABUT plan. These were taken slightly off‐isocenter, on a leaf pair, not between leaves. The additional structure in the profile can be seen. A more complicated MLC parameterization is required to mimic this structure in the planning system. Three images of the distribution are shown to highlight the increased complexity: a model with simple MLC offset, a model including corner leakage, and the measured distribution. (Representative profile for the Vendor distribution can be seen in Fig. 1. The lack of corner leakage in the Vendor model results in virtually identical profiles at both isocenter and one‐half leaf offset.)

Figure 2 plots another set of 3ABUT profiles. These profiles are taken in the same direction in the same distributions, but instead of travelling through isocenter, these profiles are shifted one‐half leaf width perpendicular to the leaf travel. The profiles in Fig. 1, due to their position at isocenter, fell in between the central two leaves of the MLC, whereas the profiles in Fig. 2 fall directly on top of an opposing pair of leaves. The two measured profiles at slightly different locations in the dose plane are quite distinct. While the profile in Fig. 1 contained simple maxima, the profile in Fig. 2 displays maxima in the same position with inset minima. An image of the 3ABUT distribution is shown to reveal the complexity along the match‐line of the segments. It was seemingly not possible to model this type of complexity in the planning system by simply adjusting the leaf offset and leaf tip leakage parameters. Instead, it was necessary to employ the corner‐leakage model to obtain a similar distribution and profile structure. Both the profile and an image of the distribution for this model are displayed, along with an image the distribution calculated using the Vendor parameter set, which does not employ corner leakage, for comparison.

**Figure 3:**
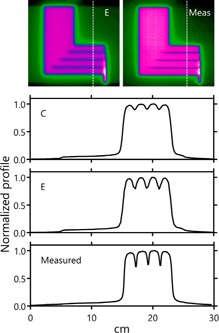
Normalized profiles taken through the FOURL distribution. The depth of the dose minima at the leaf edge junctions requires a model with a much larger groove width than the default setting if a match to the measurement is desired. However, even with this increased groove width the measured distribution cannot be matched with respect to the sharpness of the minima.

In addition to parameters describing the physical and dosimetric qualities of the leaf tips, there also exists a parameter in the model to account for tongue‐and‐groove effects. Figure 3 displays normalized profiles across the relevant leaf‐match lines of the FOURL field. The first profile exhibits the tongue‐and‐groove effect on the distribution when the default groove width parameter value is maintained. The profile differs considerably from that measured using EDR2 film. To obtain a profile that more closely matches that seen in the measurement, the groove width was increased significantly. The depth of the profile minima was matched through this adjustment, but the sharpness of those minima could not be mimicked. Images of the FOURL distributions, both calculated and measured, are displayed for comparison.

**Figure 4:**
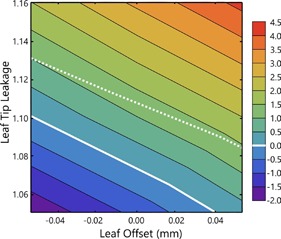
A contour plot of average point‐dose deviations between planned and measured doses in the high‐dose/low‐gradient region of the plans. The values of the leaf offset range from −0.05 to 0.05 mm, and the values for the leaf tip leakage range from 1.05 to 1.16. A level curve of parameter sets that show 0% deviation for the target dose is displayed as a solid white line. The dotted line represents the 0% level curve for the low‐dose measurements.

To investigate the effect changes to the parameter sets have on clinical plans, each of the 10 plans created using the modified TG‐119 suite dose distributions were first calculated for several MLC parameter sets where only the leaf offset and leaf tip leakage parameters were changed. In Fig. 4, the average deviation of the calculated point doses from those measured in the high‐dose regions of the dose distributions are plotted as a function of both the leaf offset and leaf tip leakage parameters. Due to the number of fields in each plan, 51 individual point doses contributed to each average value. The algorithmic nature of the calculated dose produced a consistent relationship between the two parameter values, displaying a theoretical level curve of parameter values that provided, on average, 0% deviation from measured dose. In Fig. 4, only the high‐dose point‐dose measurements are used in the contour plot; however, a similar analysis of the low‐dose point‐dose measurements produces a similar set of contours. The level curve describing 0% deviation of the low‐dose point‐dose measurement is plotted in Fig. 4 as the dotted white line. It is noticeable that the two level curves do not cross, suggesting that adjustment of only the leaf offset and leaf tip leakage parameters would not allow for simultaneously accurate in‐target and out‐of‐target dose calculation. It is of note that parameter sets that produced very good point‐dose matching also produce 3ABUT profiles in Fig. 1 (model B) that are wildly different from those measured.

**Figure 5:**
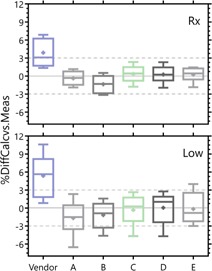
Tukey box and whisker plots of the point‐dose measurements in both low‐ and high‐dose regions of the plans for several sets of MLC parameters. ‘+’ represents the mean, box bounds the inner quartile range (IQR), and the whiskers bound 1.5 * IQR or extremum value, whichever is nearer the IQR. Any points outside 1.5 * IQR are displayed as open circles. Table 1 displays the parameters associated with each model.

The overall effect of parameter set choice for these pseudoclinical plans is presented in 5, 6. Figure 5 presents, for a series of MLC models of increasing complexity, the point‐dose measurement results for both the high‐ and low‐dose regions of the distribution.

**Figure 6:**
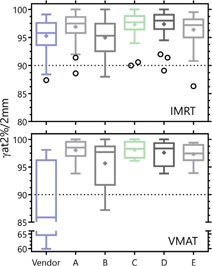
Tukey box and whisker plots of the gamma pass rates of the plans for several sets of MLC parameters. Criteria set to 2% global max dose/2 mm, 10% threshold. ‘+’ represents the mean, box bounds the inner quartile range (IQR), and the whiskers bound 1.5 * IQR or extremum value, whichever is nearer the IQR. Any points outside 1.5 * IQR are displayed as open circles. Table 1 displays the parameters associated with each model.

The vendor‐provided model displayed a surprisingly large average deviation between measured and calculated point doses, particularly so in the high‐dose region. The separation between 0% deviation in‐target and 0% deviation in the low‐dose regions that was seen in Fig. 4 was overcome in models C and D by adjusting the leaf transmission parameter. The most complex model — model E — was created by adjusting essentially all parameters to best match both the QA distribution shape and the point‐dose measurements.

Figure 6 shows the gamma pass rates for the clinical plans as measured with the MapCHECK. The gamma criteria in the plot are 2% global maximum dose/2 mm, with a 10% threshold. It is interesting to note that, despite the point‐dose deviations in target, the vendor‐provided model succeeds in providing an average gamma pass rate of >95 for all fields of the IMRT plans measured. While the model fared less well for the VMAT plans, the fact that a significant number of plans had “acceptable” pass rates at 2%/2 mm was surprising, given the point‐dose measurements. It was further somewhat surprising to see that model E — the model that most closely matched the QA fields — did not provide the highest gamma pass rates. It should be noted that at a gamma criteria of 3%/3 mm, the pass rates were practically indistinguishable for all models.

Table 2 displays the results of the IROC H&N phantom external evaluation. Each model passed external evaluation, with varying degrees of success. The results of the external evaluation in large part track with those seen in our own internal investigation, displaying acceptable gamma pass rates for all models despite fairly significant point‐dose differences from the vendor model. It is also interesting to note the relative inaccuracy of the calculated OAR point doses for the highly tuned model.

**Table 2:** IROC external credentialing results for each MLC model. Criteria for gamma is 7%/4 mm with >85% of points passing considered acceptable. Criteria for in‐target, point‐dose passing is 0.93–1.07 where the ratio is measured/calculated dose. Point doses were reported to IROC as the mean dose to the TLD.

	*γ Pass Rate*		*Primary PTV*				*Secondary PTV*		*OAR*	
	*Axial*	*Sagittal*	*Sup/Ant*	*Inf/Ant*	*Sup/Post*	*Inf/Post*	*Sup*	*Inf*	*Sup*	*Inf*
Vendor	99.9%	99.4%	0.94	0.95	0.95	0.95	0.97	0.95	0.87	0.84
A	99.9%	100%	0.99	1.00	1.00	1.01	1.00	0.98	0.99	0.97
C	99.9%	100%	0.98	1.00	0.99	1.00	1.00	0.98	1.00	0.98
E	99.9%	100%	0.98	0.99	1.00	1.01	0.99	0.98	0.93	0.91

## IV. DISCUSSION

The MLC model available in Monaco is robust and capable of providing accurate dose calculations for IMRT and VMAT plans at reasonable clinical Monte Carlo calculation parameters. The vendor‐supplied ExpressQA package can be a valuable tool to assist in model parameter determination, but it appears that tuning based on point‐dose measurements is necessary to ensure the greatest plan accuracy. The vendor‐evaluated ExpressQA procedure produced an MLC model that was noticeably inferior to that found with significant internal testing; however, despite this, the vendor‐provided model passed IROC H&N external evaluation.

The discrepancy between the vendor‐provided model and that determined in‐house most likely has a diffuse explanation, partially associated with our choice of QA equipment and partially associated with the natural limitations of dose distribution comparison as a method for tuning a head model.^(12)^ The spatial resolution of the ArcCHECK makes the visibility of small structure in the distributions difficult, and due to the higher physical density of the device with respect to water, the absolute calibration of the device requires careful consideration in Monaco with regard to dose‐to‐medium, dose‐to‐water, and effective relative electron density assigned to the image‐set.^11^, ^13^, ^14^, ^15^ These additional considerations need to be carefully coordinated with the vendor, and it is likely that a simpler QA system would provide less room for unintentional inaccuracies in head modeling. It is likely that the vendor‐assisted procedure could have provided a parameter set that produced calculated point doses closer to those found in ion chamber measurements if we had chosen a water‐equivalent QA system initially. However, given the lack of ion chamber measurements in the ExpressQA package, the reliance on using the absolute calibration of such a QA system remains a concern, and the use of planar comparison metrics with a single pseudoclinical field in the ExpressQA package places limits on the generalizability of any statements of head model accuracy using this methodology.^12^, ^16^

In general, a potential deficiency associated with the vendor‐suggested procedure for MLC modeling is the very small number of clinical fields included in the vendor package. A single dynamic dose distribution is provided, and although the field delivers a complex H&N‐style distribution, it remains a single measurement. Our in‐house testing showed that the least accurate model overall could, for any given field, have the highest pass rate at 2%/2 mm. It is not possible to obtain an accurate statistical sense of the MLC model performance without many fields to build those statistics. In fact, the major criticism of the study presented here is almost certainly the limited number of plans calculated and compared. This number was chosen partially due to the number of structure sets available in the TG‐119 package, and partially due to the extended calculation times of the Monte Carlo algorithm, which provided a somewhat ambiguous definition of what constituted a ‘reasonable’ number of model iterations and planned dose distributions.

While the QA fields are undoubtedly helpful, they are, by construction, pathological — in the sense that they display intentionally nonclinical characteristics to isolate specific aspects of the MLC. The Monaco MLC model, though robust, in many ways does not attempt to physically replicate the MLC, and as a result there are some aspects of the model which are less dosimetrically representative of the MLC than others.^3^, ^17^ In this sense, although it is tempting to endeavor to match QA profiles as exactly as possible, it may not be the case that such a model will be the most accurate for clinical calculations. Our results show this to be the case, if only marginally, and perhaps partially as a result of the inability of the MapCHECK to resolve differences in the distributions. For model E, the results of the OAR point‐dose comparisons in the RPC phantom seem to indicate that dose traversing the respective transmission regions of the MLC model can sum inaccurately at different out of field points if a very exact matching of the QA fields is pursued. The models which provided the most accurate calculations with respect to both point‐dose and planar measurement — models C and D — relied only upon adjustment of the leaf offset, leaf tip leakage, and leaf transmission parameters to match point‐dose measurements with little regard for QA field matching, tongue‐and‐groove effect, or corner leakage. Nevertheless, the lack of QA field matching was seemingly at least somewhat predictive for model B, which, despite producing reasonably accurate point doses, had the poorest gamma pass rates of the in‐house‐determined models.

The MLC model used in Monaco 5.0 uses a stack of 11 probability filters, where each filter contains a 2D representation of the MLC leaves.^3^, ^17^ In this 2D representation there are a number of parameters associated with physical aspects of the MLC that are not user adjustable. For example, the groove width parameter can be adjusted to approximate the tongue‐and‐groove effect, but there is no groove height or groove attenuation parameter for adjustment. The MLC leaves in the Agility head have a tongue‐and‐groove linkage so small that it effectively does not exist dosimetrically. Instead, the leaves are intentionally defocused, presenting a pseudo‐tongue‐and‐groove effect. Based on our measurements, it appears that the effect is not well represented by the current MLC model design, but it is most likely the case that simple Agility‐specific tweaks to the attenuation properties of the groove in the model can create better agreement. A similar situation exists for the corner leakage model, where small tweaks to the allowable length of the corner could provide better agreement. However, based on our results using less detailed MLC models, and the clinical necessity of using 3 mm voxel sizes during calculations, it could be argued that such detailed modeling would be clinically irrelevant, and potentially lead to the implementation of less accurate models through misplaced persistence.

### A. Potential recipe for MLC modeling

The process of creating an MLC model in Monaco can range from taking a few measurements to send to the vendor all the way to calculation and comparison of hundreds of point doses and dose distributions. This study indicates that, with the current model, there may be diminishing returns beyond the adjustment of three parameters: leaf offset, leaf tip leakage, and leaf transmission. As such, a reasonable procedure for the creation of an MLC model in Monaco could be described as follows:

1. Begin with the default model and create, using TG‐119 or similar pseudoclinical patient data, several different IMRT plans for sites that will typically be treated clinically.
2. Deliver the plans to a QA device that allows point‐dose measurements in both the target and low‐dose regions.
3. Compare average point doses to determine if the average dose in the target should be lowered (decrease leaf offset/leaf tip leakage to match) or raised (increase leaf offset/leaf tip leakage to match).
4. Deliver 3ABUT from the ExpressQA package to the highest‐resolution QA device available; compare result to calculated distribution using the new leaf offset/leaf tip leakage parameters. Make sure the combination chosen gives a reasonable matching to the 3ABUT delivered. If deeper minima in the profile are required, decrease leaf offset while increasing leaf tip leakage to compensate for absolute dose.
5. Once target point dose is matched with reasonable 3ABUT matching, compare average point doses out of target. If low, adjust leaf transmission up to increase out‐of‐field dose, decrease leaf offset/leaf tip leakage to compensate for in‐target increases. If high, adjust leaf transmission down, increase leaf offset/leaf tip leakage.
6. Once both in‐target and out‐of‐target point doses match and 3ABUT is reasonable, create QA plans by recalculating the standard clinical plans on a water‐equivalent phantom in Monaco (to avoid high density uncertainties) and ensure that gamma pass rates are as expected.

## V. CONCLUSION

The MLC model in Monaco is robust and can be tuned extensively to produce accurate calculated dose distributions. However, the number of tunable parameters potentially allows for an overmodeling of the MLC, and in its current form the model adjustment can be limited to changing the leaf offset, leaf tip leakage, and leaf transmission parameters to obtain a good result. The vendor‐provided ExpressQA package is very useful to check the reasonability of the model at MLC segment match‐lines, but point‐dose measurements and planar QA for several different simulated clinical plans seem necessary to guarantee a clinically accurate model.

## ACKNOWLEDGMENTS

The authors would like to thank Anthony Doemer from Henry Ford Health System, Detroit, MI, for allowing the use of his H&N SIB and Prostate+LN structure sets in this study.

## COPYRIGHT

This work is licensed under a Creative Commons Attribution 4.0 International License.
